# Covid-19 and Crisis-Prompted Distance Education in Sweden

**DOI:** 10.1007/s10758-020-09470-6

**Published:** 2020-09-02

**Authors:** Nina Bergdahl, Jalal Nouri

**Affiliations:** Computer and Systems Sciences Department, Stockholm University, Stockholm, Sweden

**Keywords:** Covid-19, Distance education, GDPR, School closure, Sweden

## Abstract

This study represents the first research effort to explore the transition from traditional teaching into distance teaching in Swedish schools enforced by covid-19. Governments made gradual and injudicious decisions to impede the spread of the pandemic (covid-19) in 2020. The enactment of new measures affected critical societal functions and included travel restrictions, closing of borders, school closures and lockdowns of entire countries worldwide. Social distancing became the new reality for many, and for many teachers and students, the school closure prompted a rapid transition from traditional to distance education. This study aims to capture the early stages of that transition. We distributed a questionnaire to teachers’ (n = 153) to gain insights into teacher and school preparedness, plans to deliver distance education, and teachers’ experience when making this transition. Results show that the school preparedness was mainly related to technical aspects, and that teachers lack pedagogical strategies needed in the emerging learning landscape of distance education. Findings reveal four distinct pedagogical activities central for distance education in a crisis, and many challenges faced during the transition. While preparedness to ensure continuity of education was halting, schools and teachers worked with tremendous effort to overcome the challenges. Results expand on previous findings on school closure during virus outbreaks and may in the short-term support teachers and school leaders in making informed decisions during the shift into distance education. The study may also inform the development of preparedness plans for schools, and offers a historical documentation.

## Introduction

Sudden and unforeseen changes to society may have a significant impact on critical functions and services. In the wake of covid-19, many people found themselves in quarantines, working and studying from home (MacKenzie 2020). The news was continuously updating on the events, informing of deaths, imposing on restrictions and limiting civil rights, which also could have increased the hamstring of food and supplies (Bayham and Fenichel 2020). After overcoming the initial chock, many governments decided that central functions in society, like compulsory education, should make a shift into distance education (Zhang et al. 2020). By March 18,107 seven countries had made country-wide decisions to close school impacting 861,737,696 learners (UNESCO 2020). Previous research on school closure (as an effect of government response to halt the spread of epidemic diseases), and the requirement of transitions into distance learning, has shown that schools interpret the new conditions in varied ways, indicating that officials need to prepare for transitions and communicate guidelines with clarity (Klaiman et al. 2011). To date, Swedish teachers have not had any previous experience of pandemics and school responses to build on. While research highlights how critical it is for the educational sector to have preparedness plans to ensure safe and functional education in times of crisis (Faherty et al. 2019; O’sullivan et al. 2009; Olympia et al. 2005) teachers and decision-makers cannot wait until the outbreak is over to make decisions. It is imperative that information is distributed in a timely manner in order to be useful.

This study aims to capture teachers experiences in the very early stages of the crisis-prompted transition into distance education. By investigating teacher preparedness and choices when implementing distance education, including the challenges they face, we hope that the study may support teachers and decision-makers in Sweden, and elsewhere, in making informed decisions to aid the transition into distance education as well as developing preparedness plans for future pandemics. The ambition is also that this study may serve as a historical document that reflects this unprecedented event. With this intention the study raises the following four research questions:

- How are teachers and schools preparing the transition into distance education?
- What digital tools are used to meet the needs of distance education in extra-ordinary situations?
- What are the pedagogical activities that distance education in extra-ordinary situations require?
- What are the challenges of distance education in extra-ordinary situations, as identified by teachers?

## Background

### Pandemic Flues from an Educational Perspective

#### Previous Pandemics

Pandemic flues have become a recurring threat to everyday life in the twenty first century, with the Bird flu, Swine flu (H1N1), foot-and-mouth disease, SARS and Zika (Nikiforuk 2008). The current spread of covid-19 includes a record number of schools and learners affected. Unlike previous flues UNESCO estimates (March 18) that the current state of school closure affects a record number or some 49% of the learners at pre-primary, primary and secondary levels of education worldwide, due to attempts to slow the spread of the virus (UNESCO 2020). Executing nation-wide school closure and an unprepared transition into digital education, for the whole school sector, implies that challenges will arise and need to be overcome without delay. While, teachers have been required to use digital technologies to initiate and facilitate at-home learning to prevent the spread of previous outbreaks (e.g. SARS, H1N1) (Fox 2003, 2007; Woodhead and Kennedy 2010), the impact across the globe, and educational levels are unparalleled.

#### Distance Education and Crisis-Prompted Temporary Distance Education

The closing of schools due to covid-19 have raised uncertainties and disagreement about what and how to teach, (Wang et al. 2020). While this crisis-prompted transition into distance education technically could be classified as traditional distance education, researchers have forward suggestions to not use the term (e.g. Hodges et al. 2020). The argument follows that there are differences in quality expectations due to limited planning, technological aspects like accessibility, security and copyright, and learning outcomes. To recognise the intra-period education responses, we aim to identify the early education responses marking the transition into crises-prompted temporary distance education. We choose the pre-fix “temporary” to discern the phenomena from traditional distance education, as practices either return to “normal”, or will transform into regular distance education, during which time it is no longer a crisis-response. In essence, distance education is characterised as remote teaching and learning - where the learner is physically separated from the teacher (Rumble 2019) while participating in a planned learning activity (Holmberg 2005). For the purpose of this article, and similarly to Raes et al. 2020, we also recognise modes of blended learning that combine distance education with traditional teaching. For example, a classroom that connects both on-site students and remote students during synchronous teaching, is referred to as a hybrid classroom, and related teaching practices as hybrid forms of teaching.

### The Situation in Sweden

#### Nationwide Educational Crisis Response

Current research has noted that the Swedish government employed a “rather relaxed” attitude toward the corona-outbreak; “encouraging” people to respect the 1.5–2 m social distance, to stay home if feeling unwell, when other countries enforced a full shutdown (Andersen et al. 2020; Qi et al. 2020). However, when the decision to close schools, nationwide, was made in Sweden, this only affected upper secondary schools (UNESCO 2020), and primary schools were left to make their own decisions based on their own interpretation of the situation. Perhaps, it was due to the decentralised decisions, and due to the pandemic being framed a health and economy crises s (Danielsson et al. 2020) that no one took the responsibility to investigate and provide a reliable overview of the extent of the crisis-response and transition into temporary distance education. The lack of information of the current state of education was for example reflected in news reporting on (primary) schools being both kept open (e.g. Dagens Nyheter 2020) and closed (SVT Nyheter 2020), and the department of education dismissing that there could be a need for hybrid teaching solutions, that as schools should remain open, and sick children should stay home without schooling (Olsson 2020b). Hence each school had to decide on how to respond to the crisis, and without proper guidelines, it was often up to the individual teacher to find pragmatic solutions in the reality they faced.

#### Digital Technologies and Digitalisation

In 2016, 98% of the Swedish students used the Internet daily, and 41% of the Swedish adolescents owned multiple devices including tablets, computers and mobile phones (Internetstiftelsen 2016) and most Swedish (and European) schools have digital strategies and agendas (European Commission 2019; The Swedish Department of Education 2017). Un-related to the covid-19 situation, the Swedish government expanded the right to adopt remote and hybrid forms of teaching and learning during specific circumstances, for example, one type of remote teaching manifests when students sit in one class room and the teachers connects from another physical location, (as a response to the lack of teachers in certain subjects in certain rural areas), or a type of hybrid teaching, where the teacher synchronously instructs online and on-site students, (as a response to educational needs for student whose absenteeism are related to physical or psychological illness) (The Swedish Department of Education 2020a, b).

Thus, during the time of the covid-19 outbreak, primary and secondary schools in Sweden were already seeing different modes of blended learning in emerging hybrid classrooms. Perhaps, it would seem like, from a knowledge and access perspective things “were in place”, and the “only” remaining obstacle was to test the capacity of the national IT infrastructure when Sweden would “go online” for work and education (The Swedish Post and Telecom Authority, 2020). However, Scandinavian research on hybrid teaching solutions had thus been ongoing for prior to covid-19 (Børsting et al. 2019; Culén et al. 2019). Such research build on well-established theories of learning, in which the learner is viewed as socially situated in a community of other learners, and there engage in varied learning activities that enable polysynchronous interaction such as, planning, conceptualising,, re-conceptualisation, peer modelling, reflection, feedback and scaffolding (Dalgarno 2014; Hattie 2009; Laurillard and Derntl 2014; Lave and Wenger 1991).This initial research points out that students with long-term illness and social isolation may benefit from being socially included in the class through the use of digital technologies (No Isolation 2020). Research constantly highlight that the social aspect of learning with technologies are critical for engagement (Bergdahl et al. 2020). Despite potential, emergent research on effects of the hybrid teaching thought to redeem social isolation, revealed that the students did not always feel socially included (Svensson 2019), which indicates that merely employing technical solutions may not be sufficient to redeem a problematic situation. This initial research points out that students with long-term illness and social isolation may benefit from being socially included in the class through the use of digital technologies (No Isolation 2020). However, during crisis-prompted temporary distance education, teachers might not have the time, insight or preparation to re-design or adjust their teaching in accordance with online requirements for effective community supportive learning.

#### Discrepancy Between Institutional Response and Digitalisation of Education

Reviewing the intra-period debate, there seems like there was at least two separate understandings of the current situation in Sweden. On the one side, a bill acknowledging hybrid and remote forms of teaching had been passed (The Swedish Department of Education 2020a, b), and received critique for already being outdated, with demands raised for a more modern legislation that would enable distance education, post corona (Skolvärlden 2020), simultaneously as the teacher union highlighted the covid-19 solutions forced their member (teachers) to work twice as hard due to hybrid teaching forms (Olsson 2020a). On the other hand, the Swedish National Agency for Education stated that schools should not offer hybrid solutions, as there was no need as schools should remain open and sick students should stay at home and receive no schooling (Olsson 2020b). Not only until June 30th, after the covid-19 affected semester ended, the educational department updated their website acknowledging the hybrid classroom, and added that teachers with symptoms were allowed to engage in remote teaching from home (The Swedish National Agency for Education, 2020).

### Emerging Responses to Tackle the Covid-19 Impact on Education

Schools can learn from previous events to sustain continuity of education better. Research have repeatedly pointed to a need to develop preparedness plans for education in order to sustain schooling in times of crisis (Faherty et al. 2019; O’sullivan et al. 2009; Olympia et al. 2005). However, during the SARS outbreak in 2003, research reported that teachers felt unsure about how to use digital technologies in education (Fox 2003). For instance, few teachers expressed they did not know how to re-think their strategies even though digital technologies had been continuously used for learning. The study identified key factors that could have eased the transition from traditional teaching into crisis-prompted distance education was: (1) on-the-spot technical support, (2) teachers’ pedagogical strategies for distance learning and (3) students being introduced to distance modes of teaching and learning and related collaboration strategies (Fox 2003). While specific experiences of educational technology from the SARS outbreak might not be relevant to inform teachers of today a general need of supportive structure and a lack of guidelines and policies identified then (Cauchemez et al. 2009; Fox 2003, 2007) may yet be relevant today.

## Method

During the outbreak of the covid, many teachers were required to make a shift into distance education. The researchers followed lively discussions in media and teacher online forums and decided to capture this transition in a timely manner by distributing a questionnaire in six Swedish Facebook groups used by teachers. We employed snowball sampling, to reach as many teachers in as short time as possible, to maximise insights. To achieve this, we shared the linked in online forums, and promoted teachers to share the link with colleagues. To target relevant online forums, we selected all Swedish Facebook groups, that in their title indicated there would be members affected by the transition: “Distance education in Sweden”, “ICT tools”, “ICT pedagogy and School development”, “ICT for English teaching”, “Year 4–6 tips and ideas”, “iPads in pre-schools and schools”. We posted a brief description of the purpose and scope of the study, which was to promptly provide teachers with an overview of the current situation. To enable this, we gathered data between the 19th and the 27nd of March 2020. While most Facebook groups were already established, we found that we received the most response (in the commentary field) from the group “Distance education in Sweden”. This group Facebook group created on March 15, had no fewer than 17,000 members after two weeks, with teachers seeking collegial support to response to the crisis of covid-19 (“Distance Education in Sweden,” 2020). Teachers were also encouraged to share the link to the questionnaire in their schools. The questionnaire did not survey from which forum of distribution the teachers had accessed it. However, we included an option to disclose the geographical location. The 153 teachers, were working in 14 cities across Sweden: Alingsås, Falköping, Göteborg, Kristianstad, Linköping, Malmö, Norrköping, Stockholm, Södertälje, Trosa, Torsås, Uddevalla, Umeå and Vetlanda. The questionnaire which included four background questions (see Sect. 3.1) targeted four general areas, namely (1) strategies and preparedness for implementing distance education; (2) digital tools used for distance education and what they support and their limitations; (3) pedagogical adaptations when transiting to distance education; and (4) general challenges faced when implementing distance education in crisis. The full list of questions:

- Which level are you teaching?
- What is your level of experience in distance education?
- To what extent do you have access to digital learning resources?
- To what extent do you usually teach using digital learning resources?
- Which digital learning resources are you using for distance education?
- How are you making sure that distance education is accessible for all students?
- Which functions and digital support do you need to deliver meaningful distance education?
- Which needs are met with current tools and which are not?
- Which challenges do you or your students face when using digital tools for distance education?
- Which pedagogical adaptations are you doing to transit to distance education?
- How are you and your schools preparing for distance education?
- How are you planning lessons today in relation to the school schedule?
- Do you or your school have a strategy for transiting to distance education?
- Which pedagogical activities are important in distance education in the situation created by covid-19?
- What have gone well and what bad in the transition to distance education?
- Share your best recommendations to other teachers that plan to transit to distance education

The data was analysed using descriptive statistics and thematic analysis. Both authors worked closely to conduct the analysis using Nvivo (version 11.4.3) and post-it notes. Each author analysed half of the data set. Then, in depth discussions were made with regards to the identified themes. These insights guided the structure and re-structuring of themes and sub-themes. The process is best described as iterative as discussions triggered revisiting of the analysis, and clarification of the identified themes. For example, an initial idea was that distance education seemed to be troublesome for at risk students. During in depth discussion, it became clear, that the learning landscape had transformed: that the students, and not only at-risk students, would respond in differently than during traditional teaching, and that foreseeing this change was hard. We also recognised that there was a changed in the teacher role, in the emerging learning landscape.

### Respondent Background Information

**Fig. 1:**
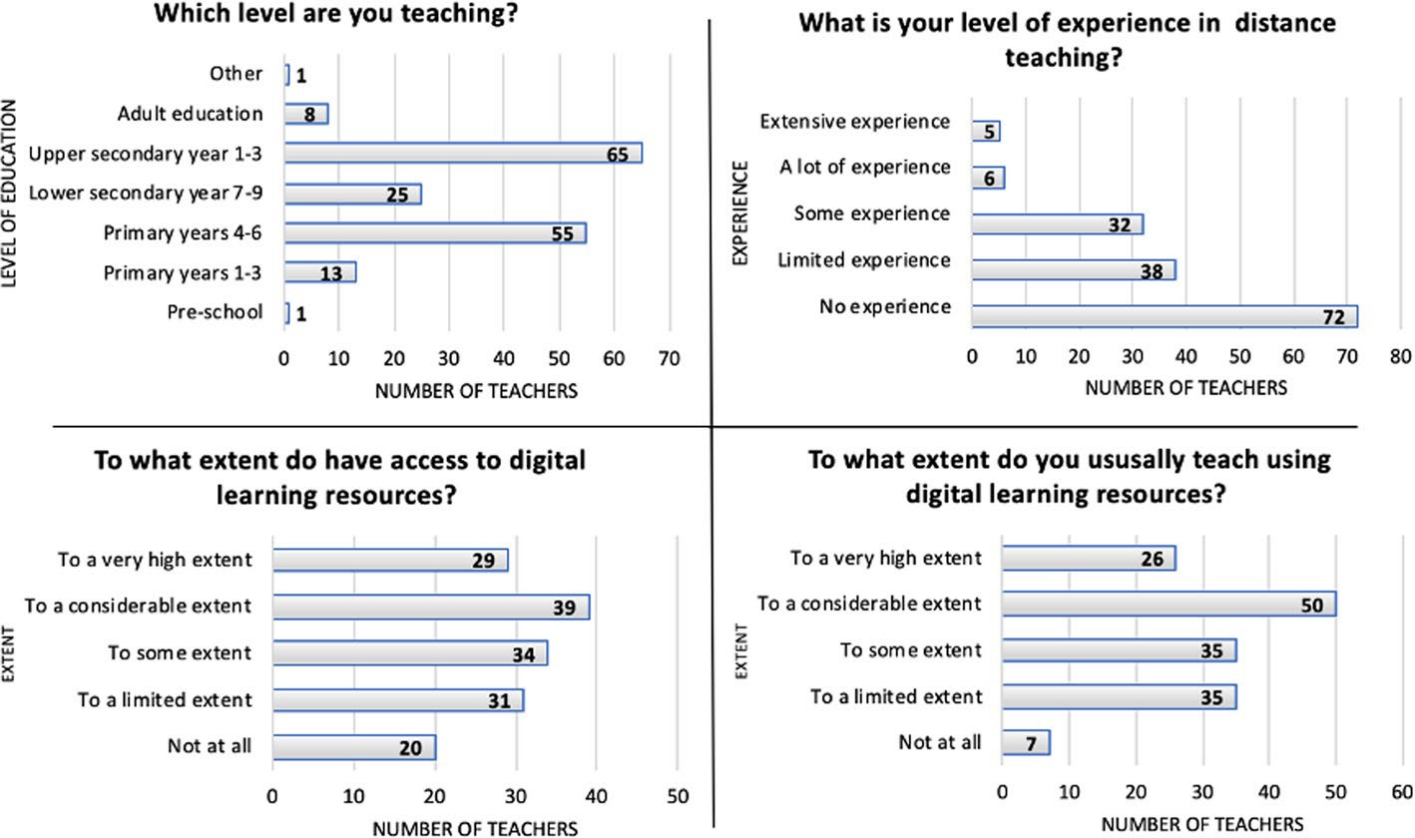
Teachers’ experience of digital technologies and distance teaching

A majority of teachers reported that they had no or limited experience in distance education (see Fig. 1). The average age of the teachers was 26 years.

About a third of the teachers said that they did not have access, or only limited access to digital learning materials, and not at all, or only to a limited extent, used digital learning materials. Noteworthily, while twenty teachers reported they did not have access to digital resources to teach their subject in their school, only one third three reported that they never used digital learning resources. This suggest that teachers either use general learning resources (not subject specific), or that they include other learning resources not provided by their school. About half of the teachers (45–50%) expressed that they both had high, or very high, access to digital teaching materials and used these accordingly (see Fig. 1).

## Results

We identified four over-arching themes: (1) School and teacher preparedness; (2) Strategies when shifting to distance education; (3) Central learning activities for distance education in a crisis and (4) General positive experiences and challenges. These themes are presented below.

### School and Teacher Preparedness

We identified two sub-themes under the category School and teacher preparedness: (1) Choosing digital technologies and considering the General Data Protection Regulation (GDPR) and (2) Mapping student access to digital technologies and school policies.

#### Choosing Digital Technologies and Considering GDPR

**Fig. 2:**
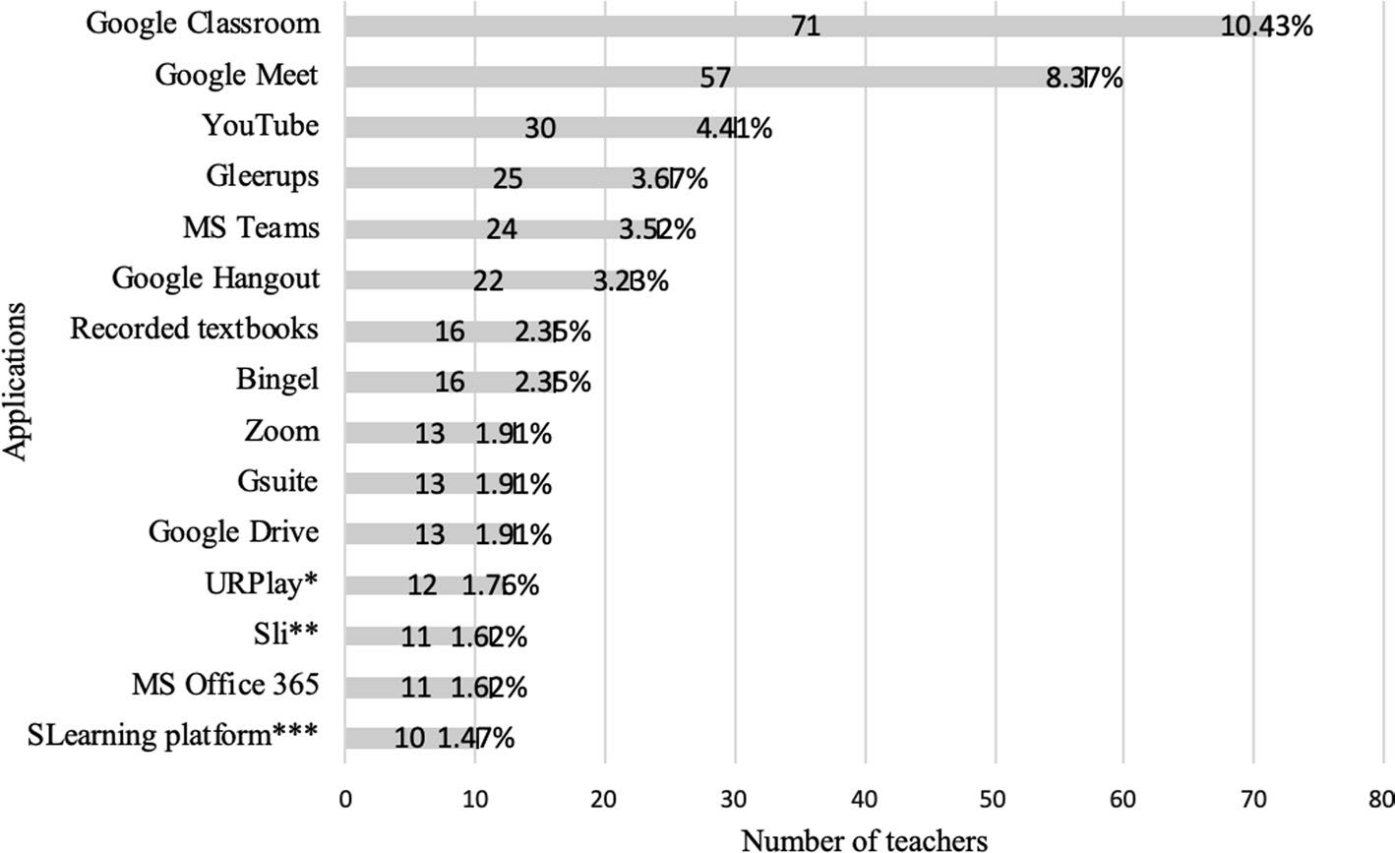
Applications chosen by teachers to enable transition into distance teaching (*URPlay is a government-supported educational play channel, ** Sli is an online repository with media and film for educational use, SLearning platform, is one of the major distributors of learning platforms (Skolplattformen))

Teachers work intensively to adapt their planned learning activities to fit distance education. Therefore, we asked the teachers what applications they use in the transition from traditional to distance education. The result was 152 different applications. Fifteen applications, dominated (see Fig. 2). A closer analysis revealed that the most common tools (and requested functionality), were applications that enabled real-time video-conferencing (Zoom, Google Meet & Hangout, Microsoft Teams), pre-recorded seminars (YouTube), communication and collaboration (Microsoft Teams, Google Classroom), sharing materials (Google Classroom, Google Drive, Microsoft Teams), and digital learning resources in Swedish (Gleerups, Bingel). Many teachers and schools take GDPR into account when choosing platforms and tools. Teachers frequently pointed out that some Google applications met the emerging pedagogical needs in a better way than other applications on the market, while also pointing out that the use of these may be illegal if GDPR agreements are not signed. Many teachers expressed frustration at the municipalities’ strict adherence to the GDPR, which enforced a use of tools and platforms with limited functionality (with which the municipalities had contracts). At the same time, many teachers seem to consider GDPR a secondary factor when selecting tools, with the justification that exceptions should be possible in a crisis if needed. This reasoning, in turn, rests on two main arguments expressed by teachers; first, that many schools, up until now, have not had a reason to secure data privacy agreements for tools and platforms suitable for distance education; and second, that the existing tools and platforms with which the municipality had contracts to use, and which were recommended for distance education did not meet the pedagogical needs. Data suggests that many schools use accessible applications despite not having GDPR agreements in place.

#### Mapping Student Access to Digital Technologies

Implementation of distance education requires that students have access to computers or tablets and internet connection. Although many students do, some students have to share one device with others in the family; other students may have a limited data plan or unstable internet connection. These factors are prerequisites for daily use of video conferencing tools. The answers from the teachers show that many schools had not yet surveyed student access to devices and internet, and were currently doing so. Schools that had identified students who could not participate online, solved this in different ways. They either invited the students to study in school, directed them to the local library for internet, or sent out printed learning materials via traditional post and matched these with telephone support.

### Strategies When Shifting to Distance Education

We identified two sub-themes related to strategies when shifting to distance education: (1) School and teacher strategies and (2) Approaches to individual work and interaction in Technology-enhanced Learning, reflected below:

#### School and Teacher Strategies

“Strategy ?!!– We got 24 h”

Asking the teachers if they had a strategy and what that strategy entailed, the majority of the teachers reported that their school had no strategy to implement distance education, less a strategy to respond to a school closure (which is quite understandable given the swift change that covid-19 implies). Some teachers reported they had allocated time to prepare with colleagues, while others expressed they were left to their own devices, with little or no support. Some teachers confirm that schools have digital strategies and agendas, but point to that policies or guidelines were outdated, or that digital strategies were not sufficient during a pandemic outbreak. Instead, teachers worked pragmatically with what they had to intensely expand on their experiences and gather new skills. The results reflect disperse and incoherent ways for schools to tackle this nation-wide extra-ordinary circumstance.

Learning becomes increasingly complex as teaching and learning transfers from the physical classroom, to synchronous and asynchronous interactions across platforms. While schools might not want to strive for a coherent approach to interaction given the variation in contexts, social distancing generates negative effects on general well-being regardless of age and context. Redeeming social isolation by through digital technologies does not guarantee a student feels socially included. Thus, social aspects of learning may impact general well-being, and may be particularly important during social isolation. This warrants the exploration of approaches of the balance of individual work and interaction in TEL. Exploring planned designs of learning, from an interaction and social aspects perspective, we identified three themes: (1) synchronous teaching and learning, (2) synchronous and asynchronous teaching and learning, and (3) asynchronous teaching and learning.

#### Synchronous Teaching and Learning

About a third of the teachers planned for continuous synchronous teaching with teacher-led lessons which included planned peer-to-peer interactions, available teachers, short instructions and individual work and feedback. When teachers shared their views on individual work and interaction in Technology-enhanced Learning, there was considerable variation in time and interaction. Time refers to the time allocated for interaction. The most common view was that students should work individually during regular lesson time.

#### Synchronous and Asynchronous Teaching and Learning

A combination of synchronous and asynchronous teaching and learning was the most common. However, some teachers had the opinion that a minimum time should be allocated for interaction; that students should follow instructions previously sent out. A combination of synchronous and asynchronous teaching here refers to the balance between individual work and synchronous peer-peer/peer-teacher interaction. Examples of synchronous and asynchronous teaching and learning, is for example when there is less teacher-student interaction (and this is not replaced with peer-to-peer interaction) than during synchronous teaching and learning. As seen in planning independent individual studies during class with results shared either at the end of the class or asynchronously.

#### Asynchronous Teaching and Learning

While a substantial number of teachers planned to emphasise individual work, about a sixth of the teachers planned for written instruction and asynchronous submission, with no interaction.

Disparities reflect that there is no coherent view on how to teach students, in terms of meeting the need for social interaction in a time of crisis.

### Central Pedagogical Activities for Distance Education in a Crisis

Four sub-themes were identified: (1) Video-based communication, (2) Distribution and sharing learning material and exercises (3) Student interaction and collaboration and (4) Assessment and examination:

#### Video-Based Communication

A majority of the teachers who had initiated distance education, expressed a need for video-based communication. They placed great emphasis on tools that enable communication in both asynchronous and synchronous ways, and expressed that digital technologies, for educational purposes, should allow for a variety of functions: that everyone can communicate between themselves, using both audio and video, in a full class, in smaller student groups and individually with the teacher. For these purposes, teachers now used tools such as Zoom, Google Hangout and Microsoft Teams.

However, some functionalities were missing. (1) Many of the teachers emphasised that they needed to be able to record introductions and instructions that students could access later. Teachers highlighted that recorded materials may assist students who miss an introduction, and may be helpful for students who benefit from repetitions and/or prefer controlling the pace. Many teachers pre-recorded learning materials to re-use. (2) In this context, the importance of being able to share educational video resources, and make these available online, was also emphasised. While pre-recorded material has the disadvantage that the interaction between teacher and student is limited and that the student cannot ask questions, it also saves the teacher’s time in the long-run. (3) The importance of discussion forums has also been emphasised by the majority of teachers, as well as (4) tools for sharing instructions with parents (when applicable). Many of the video communication tools used by teachers today include chat functions, but it was pointed out that in some applications, these chat functions only allow for synchronous communication. (5) Several teachers expressed a need for tools to also support asynchronous chat; as this would enable a later response in case the time in class was insufficient to reply to all students. Some teachers point out that text chat can be useful when video conferencing tools are lagging, crashing or when the internet connection is unstable. Thus, teachers request chat features for asynchronous communication as a supplement.

#### Distribution and Sharing Learning Material and Exercises

**Fig. 3:**
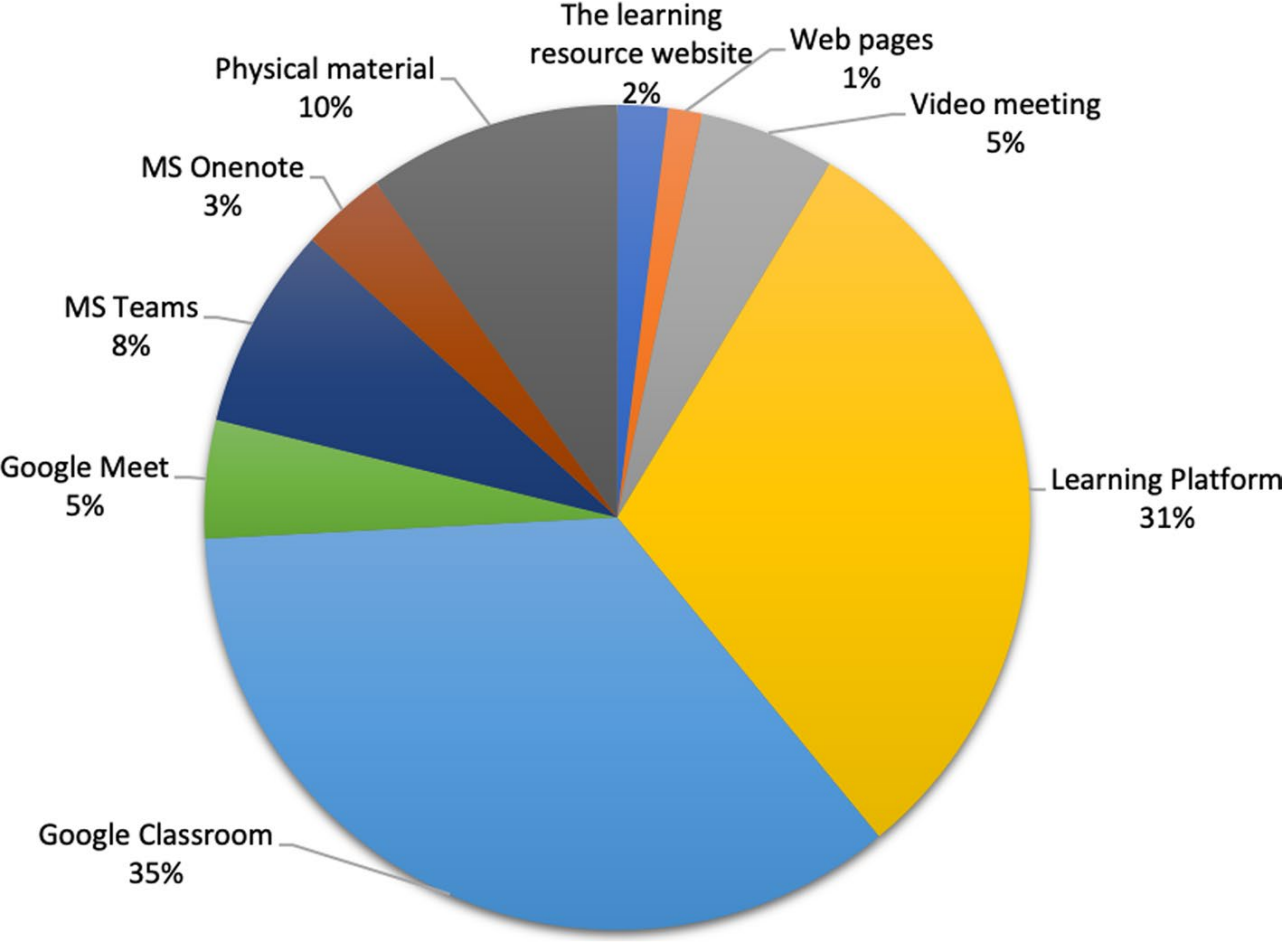
Distribution of learning materials

Several teachers say that they try to follow exiting schedules to instil a well-needed minimum level of familiarity and predictability for the students. Other teachers express that adjustments to the schedule are made, with joint start-up, some review, self-study and joint follow-up. When striving for students to recognise the lesson structure, teachers emphasised the need to share learning resources and information with the students. These may be instructions, exercises or other resources needed for learning. Many teachers used The Learning Platform (a specific learning platform: Skolplattformen), and Google Classroom, (see Fig. 3) to distribute semester planning, schedule, tasks et cetera. However, there were a number of teachers who still relied on the physical materials for students to pick up at school or that would be sent out by posts.

#### Student Interaction and Collaboration

Regarding support for collaboration, the majority of teachers consider collaboration areas to be important for students, partly because it facilitates for the teacher that the students work and help each other but also that the importance of the students’ social interaction is emphasised. It was not straight forward for most teachers how to enable student interaction and collaboration using the different digital technologies at hand (which may explain some of the diversity seen in Fig. 3). Many teachers also expressed that it would be helpful to support collaboration with other teachers for joint planning, sharing lesson material and reflection.

#### Assessment and Examination

Many teachers requested support for attendance control, which was lacking in the existing tools, especially the learning platforms and video communication tools. The teachers who had just initiated distance education used hand-in assignments and confirmatory questions to confirm attendance, and considered these satisfactory. Some teachers also asked to differentiated way to mark attendance, when students are present in the physical classroom, and when they attend via digital technologies. Teachers were insecure of how to conduct diagnostic tests in distance learning, and also expressed that semester grades might be suffering, as there was a lack of tools to support secure digital examination: that existing tools and platforms either lack such functionality or do not fully meet the actual needs.

### General Positive Experiences and Challenges

The teachers described the transition as a tremendous change, with “hysterically many things to juggle simultaneously” and well-experienced teachers express they “felt like a novice teacher”, and that they had “a job that never ended”. Despite this, the majority of teachers are positive and reported that while the technology has limitations, they worked around this and that lectures had run surprisingly well. Teachers expressed being surprised to discover better student contact and reacted positively to seeing students in their home environment. Teachers’ responses reflect discovery of a learning landscape in which social interactions and conditions for learning are not the same as in the traditional classroom; placing new demands on the role of teachers. Many teachers were surprised to discover that students may react and respond differently than in the physical classroom. For example, it was hard to foresee which students would attend distance education. Some students who had functioned well in the traditional classroom would start displaying disengagement behaviours. In contrast, teachers report that some (expected) at-risk students, i.e. school refusers, silent students, tech-savvy students, and students with autism, would increase their participation in learning activities when attending via technologies as these was seen to work in their favour. The transition to distance education revealed that the overall attendance in several teachers’ classes were higher than when requiring a physical presence. Moreover, teachers reported that students working from home could often concentrate better as they were not distracted by peers in the classroom. Many teachers were generally pleasantly surprised with the engagement, motivation, patience and technology expertise most students displayed, and expressed that the transition had gone overwhelmingly well. At the same time, there were more cautious teachers, who pointed to the novelty of the situation, and warned that adverse consequences might emerge later.

The teachers also noted several challenges. For instance, applications or connections that are unstable; that students need technical support when it comes to remembering their login details; that some students do not have the skills needed to manage the technologies effortlessly for learning, and that some students struggle to understand written instructions. Furthermore, not all students had parents to support them, and teacher experienced that providing technical support from a distance was hard. Moreover, teachers too lacked technical support. Teachers struggled with taking attendance, and were unsure of how to perform legally sound assessments and examinations. Furthermore, the shift from the traditional classroom to virtual classrooms may impede the insights into students’ learning processes, making it more challenging to identify students that need support. In addition, many teachers found that video-conferencing were fraught with challenges due to a lack of netiquette among the students (e.g. talking simultaneously, muting each other’s microphones and logging out peers, and so on). Extended hours were also identified as challenging as many students lack the patience and motivation to be seated in front of a screen for a longer period of time. At the same time, students react differently when learning from home: some students began to display distress from isolation; some displayed difficulties with motivation and discipline, saying that they missed school and friends and were distracted by concurrent activities at their homes. Teachers also expressed concerns that students might not keep track of schedule and tasks, and may participate in lessons from unfit environments (e.g. hanging outdoors with friends during a video lecture).

## Discussion

We raised the question “How are teachers and schools preparing the transition into distance education?”, and forward that while most schools have digital agendas (European Commission 2013, 2019), results indicate that the strategies needed in the covid-19 crisis are different (Anderson 2020; Centers for Disease Control and Prevention 2020; Fox 2003), which reinvokes earlier callings for school preparedness plans (Olympia et al. 2005). After all, the transition is not a well-prepared long-term shift to distance teaching, but an unprepared rapid change that risk becoming increasingly complex as it includes teaching students who may experience negative consequences of the virus and/or social isolation, i.e. distress, depression, illness or even loss. We also raise d the question “What digital tools are used to meet the needs of distance education in extra-ordinary situations?” and indeed found that school preparedness was mostly technical; i.e. focusing on available devices and applications. Yet, outdated guidelines, impeded uses that adhered to GDPR, and while Google products was the most commonly used, teachers expressed that there were many technological issues that limited their practice. Despite this, there were little reflection on pedagogical preparedness; instead results reveal disperse and incoherent ways for schools to tackle this nation-wide extra-ordinary situation. Exploring the question “What are the pedagogical activities that distance education in extra-ordinary situations require?” we found that school personnel displayed tremendous effort and creativity to develop pragmatic solutions. Wanting to support students to not fall behind in their education, many teachers took it upon themselves, during spring, to accept students to attend in class via digital technologies, and in some schools, where 50% of the students were kept home by their parents, not offering hybrid solutions was not an option. The activities that were emphasised were communication, collaboration, sharing learning materials and uploading of student work, assessment and examination. Lastly, we raised the question “What are the challenges of distance education in extra-ordinary situations, as identified by teachers?” Results revealed that many teachers lacked previous experience of using digital tools to support these activities. Thus, teachers were put into a situation that emphasised a need for certain activities, that they felt ill-prepared to deliver. Based on the views and opinions of a majority of teachers, we also conclude that teachers very quickly realised that distance education in this situation renders a new teaching and learning landscape with unique conditions and requirements, sometimes very different from ordinary classroom teaching. The new situation includes changed roles for both teachers and students. Some students who had functioned well in the psychical classroom, might start to disengage, and others were seen to benefit from attending via digital technologies and could concentrate better when working from home. Further research is warranted to in depth, investigate the implications of the *new teaching and learning landscape*.

### Preparedness Plans

While schools were not pedagogically prepared for the transition into temporary distance education, less hybrid forms of teaching, during the outbreak of covid-19, experiences gained, and lessons learned, may be gathered and collectively evaluated to form the basis of pedagogical plans, for use now and in developing preparedness plans for the future.

Based on the findings here, and other research and reports we propose that preparedness plans for schools should be developed and take critical aspects into consideration. First, social distancing requires that more people work and study from home, which in turn requires stable and high-speed internet connectivity. Thus, having a well-developed Internet infrastructure is a pre-requisite to enable the masses to work and study from home (European Commission 2013), but not sufficient. Schools need to ensure that students also have a device for educational purposes. Second, the risk of distress and depression needs to be considered, as social distancing might affect student general well-being (Faherty et al. 2019). When physical social interaction is redrawn, students may feel invisible, excluded, even when participating via online technologies, and the designs of learning activities must take this into consideration, especially during social distancing, when isolation affects all students, regardless of age. Third, conditions for special needs students need to be considered along effective strategies informing teachers on how to support special needs students during distance education. Fourth, a future school preparedness plans must include a selection of digital tools that function well for distance education—that also take these central activities into account. These activities should also be considered by developers of educational technology. The challenges many teachers face when using available educational technology, for instance, risk of breaching GDPR, a lack of secure digital examination tools or communications tools that are tailored for pedagogical activities, provide support for this recommendation. Fifth, the closing of schools due to covid-19 have raised uncertainties and disagreement about what and how to teach, (Wang et al. 2020). While it is common that frameworks applied within distance education apply theories that build on learning as a social phenomenon (Hattie 2009; Laurillard and Derntl 2014; Lave and Wenger 1991), the emergent learning landscape dramatically challenges the teaching practices when transferred to online learning environment, where learning should be orchestrated synchronously and asynchronously across multiple platforms (Dalgarno 2014). Not knowing how to exam students have also negatively impacted European students’ possibilities to re-enter a state of normal, i.e., being able to plan their (educational) future, post corona (Burgess and Sievertsen 2020), and Sweden when schools tried to support students to participate in school even when isolating with symptoms, by offering hybrid teaching and learning - such effort was deemed unnecessary by the department of education (Olsson 2020b). Future preparedness plans ought to objectively survey the long-term consequences of pragmatic practises before accepting or dismissing them.

Conclusively, as covid-19, was mainly framed as a health and economic crises (Danielsson et al. 2020), the skewed focus may have led to overlooking the need for educational preparedness plans and teacher’s professional competence to respond to online or hybrid forms of teaching. Teachers’ critical function in society during a pandemic must not be underplayed. Students may experience distress, depression or even loss, in social isolation during a pandemic. While it is essential to uphold the teaching-routines during a crisis, it is equally, or even more important to consider students’ psychological well-being. In addition, teachers experience of the transition into temporary distance education are critical to explore when developing an informed preparedness plan for Swedish education.

## Limitations

Several limitations must be considered when interpreting the results of this study. This study employed an intense data collection method under a few days to respond to the acute crisis of the covid-19 situation and transition into distance teaching for Swedish teacher. The data-collection was limited to Facebook groups and uncontrolled sharing of the link by respondents, and thus, would not access teachers excluded from those channels. Yet, the groups were large with tens of thousands of participants. However, considering that data-collection took place in the midst of an historical and dramatical pandemic, which create challenges for participant selection, we would argue that its rather cumbersome to succeed with an optimal participant selection. Moreover, this study reflects the earliest phase of the transition, and as such is not capturing how things might develop, which should be followed up with future studies.
